# Antioxidant and Anti-Inflammatory Activities of Kenyan Leafy Green Vegetables, Wild Fruits, and Medicinal Plants with Potential Relevance for Kwashiorkor

**DOI:** 10.1155/2015/807158

**Published:** 2015-07-08

**Authors:** H. R. Tufts, C. S. Harris, Z. N. Bukania, T. Johns

**Affiliations:** ^1^Centre for Indigenous Peoples' Nutrition and Environment, School of Dietetics and Human Nutrition, McGill University, Sainte-Anne-de-Bellevue, QC, Canada H9X 3V9; ^2^Department of Biology, University of Ottawa, Ottawa, ON, Canada K1N 6N5; ^3^Centre for Public Health Research, Kenya Medical Research Institute, P.O. Box 20752-00200, Nairobi, Kenya

## Abstract

*Background.* Inflammation, together with related oxidative stress, is linked with the etiology of kwashiorkor, a form of severe acute malnutrition in children. A diet rich in anti-inflammatory and antioxidant phytochemicals may offer potential for the prevention and treatment of kwashiorkor. We selected and assayed five leafy green vegetables, two wild fruits, and six medicinal plants from Kenya for their antioxidant and anti-inflammatory properties. Consensus regarding medicinal plant use was established from ethnobotanical data. *Methods*. Antioxidant activity and phenolic content were determined using the oxygen radical absorbance capacity (ORAC) assay and Folin-Ciocalteu procedure, respectively. Anti-inflammatory activity was assessed *in vitro* targeting the inflammatory mediator tumour necrosis factor-alpha (TNF-*α*). *Results. Mangifera indica* (leaves used medicinally) showed the greatest antioxidant activity (5940 ± 632 *µ*M TE/*µ*g) and total phenolic content (337 ± 3 mg GAE/g) but *Amaranthus dubius* (leafy vegetable) showed the greatest inhibition of TNF-*α* (IC_50_ = 9 ± 1 *μ*g/mL), followed by *Ocimum americanum* (medicinal plant) (IC_50_ = 16 ± 1 *μ*g/mL). Informant consensus was significantly correlated with anti-inflammatory effects among active medicinal plants (*r* ^2^ = 0.7639,  *P* = 0.0228). *Conclusions*. Several plant species commonly consumed by Kenyan children possess activity profiles relevant to the prevention and treatment of kwashiorkor and warrant further investigation.

## 1. Background

Kwashiorkor, a form of severe acute malnutrition in children, is a concern in Sub-Saharan Africa. Although the etiology of kwashiorkor remains misunderstood, proposed theories that implicate protein deficiency [1], aflatoxin contamination [2], and oxidative stress [3] inadequately explain the symptoms and biochemical evidence. Biochemical markers, as supported by a recent review, and differences in gut microbiome populations indicate that inflammation is likely a key component to the etiology of kwashiorkor [4, 5].

Children with kwashiorkor show elevated levels of inflammatory markers [6, 7], including TNF-*α* [6]. Moreover, fatty liver development, capillary membrane leak, and edema, all associated with kwashiorkor, can be potentiated by TNF-*α* [8, 9]. Bacterial overgrowth of the small intestine also has been documented in affected children [3], which can lead to increased TNF-*α* production in the liver [10]. The role of the gut microbiome in kwashiorkor was recently assessed by Smith et al. [5], who found that children with kwashiorkor had an overabundance of bacteria associated with inflammatory bowel disease and inducers of a proinflammatory T helper 1 response in their fecal microbiota. Therefore, a link between the gut microbiome and the inflammatory state seen in children with kwashiorkor appears likely.

As observed in many other diseases, inflammation and oxidative stress are mutually implicated in kwashiorkor. Antioxidant supplements have been previously used in treating and preventing kwashiorkor on the basis that oxidative stress was the primary underlying cause. However, the effectiveness of antioxidant supplements in treating or preventing kwashiorkor is debatable. Providing antioxidants to children with kwashiorkor through their diet improves survival [11, 12] but the use of antioxidant supplements does not appear to be effective as a preventative intervention [13]. Health benefits from antioxidants are more likely through synergistic interactions between nutrients taken in the diet, specifically the phytochemicals in vegetables and fruits [14], which are usually not included in antioxidant supplements. Whereas supplements are important for correcting nutritional deficiencies, long-term health benefits are more likely achieved through focusing on specific foods, nutrient interactions, and a diverse diet [15]. Additionally, if inflammation is the primary root cause, antioxidants would not address the underlying inflammatory condition. Therefore, the consumption of anti-inflammatory compounds and antioxidants through a diverse diet including whole foods may be more effective for prevention and treatment.

Leafy green vegetables (LGV) and fruits are important sources of polyphenols, which possess both anti-inflammatory and antioxidant properties [16, 17]. Moreover, wild-harvested plants, including LGV species indigenous to Sub-Saharan Africa, are often richer than exotic cultivars in terms of both polyphenol and nutrient content [18]. In Sub-Saharan countries, LGV represent vital components of the diet, notably for children during famine and drought, as many traditional cultivars and wild plant species are better adapted to local ecological conditions [19]. In addition, the use of plants for both food and medicine overlaps in many cultures in Sub-Saharan Africa [20], suggesting that medicinal plants already contribute to the diet of children.

With over 7100 documented plant species and more than 220 traditional LGV growing wild in Kenya [21] where kwashiorkor is an ongoing concern, this study sought to identify traditionally consumed LGV, wild fruits, and medicinal plants within a rural Kenyan context and characterize their antioxidant and anti-inflammatory properties. We selected materials for study based on dietary and medicinal data from interviews conducted with mothers in Makueni County, Eastern Kenya, on the plants most commonly eaten and/or used by their children. To the authors' knowledge, no studies to date have assessed antioxidant and anti-inflammatory properties in plants based on their potential relevance for kwashiorkor.

## 2. Materials and Methods

### 2.1. Dietary Interviews

Plants for investigation were selected based on interviews of mothers who had at least one child under the age of five years. The mothers were randomly selected from household lists provided by the Assistant Chiefs from five different villages within Kaiti Division, Makueni County, Kenya. In total, 50 informed and consenting mothers, ten from each village, were interviewed from June to August 2011. Ethics approval was obtained from the Research Ethics Board of the Faculty of Agricultural and Environmental Sciences at McGill University, Montreal, Canada. The researcher had local approval through affiliation with the Kenya Agricultural Research Institute (KARI).

A Food Frequency Questionnaire (FFQ) was used to gather dietary information about the child's intake of food items over the past seven days and throughout the different seasons (for vegetables and fruits only). The FFQ listed a total of 89 food items based on focus group data the researchers collected and a food item list developed by Bioversity International of wild foods from Kitui County, approximately 100 km from Makueni County. Twelve traditional LGV and twenty-five wild fruits were included in the FFQ.

### 2.2. Ethnobotanical Survey

Data about medicinal plants the mothers knew of or used for treating illnesses in children less than five years of age was gathered with a traditional knowledge/ethnobotanical survey that included a list of open-ended questions about malnutrition, kwashiorkor and its cause and treatment, plants used to treat malnutrition, and commonly used medicinal plants given to children. In addition, mothers were asked if they knew of plants used to treat eight specific illnesses or symptoms affecting children under five years (i.e., malaria, diarrhoea, pneumonia, swelling, flu/fever, skin diseases/rashes, inflammations, and additional GI tract symptoms).

These illnesses were queried due to the inflammatory cause of some and also to identify plants used for paediatric purposes which are likely to be safe, culturally appealing, and accessible. The participants were asked what part of the plant was used, what the preparation methods were, what the dose amount was, and how it was administered [22].

### 2.3. Consensus

A consensus analysis was modified from methodology developed by Leduc et al. [23]. Consensus takes into account (1) the number of different symptoms the plant is used to treat and (2) how frequently the plant is cited for any symptom by different correspondents, in this case the mothers [23]. The parameters for consensus are expressed in the following equation, modified from Leduc et al. [23]:

(1) $$\begin{array}{c} \text{Consensus}=\frac{\left(\sum s\right)+\left(\sum f/F\right)}{2S}. \end{array}$$

In the equation, *s* is the symptom contribution for the plant, which is calculated for each symptom with *s* = 0 if the symptom is not treated and *s* = 1 if the symptom is treated by the plant. *F* is the total number of mothers who provided ethnobotanical information (*F* = 46). *S* is the total number of symptoms (*S* = 8 symptoms specifically asked about plus 22 additional symptoms mentioned = 30). *f* is the citation frequency for the plant by all correspondents, which is calculated for each informant as *f* = 0 if not cited and *f* > 0 if cited to treat one or more symptoms. The sum of *f* values would be the total citation frequency where the maximum total would be equal to *S* × *F* [23].

### 2.4. Plant Collection and Processing

The most commonly consumed traditional LGV, wild fruits, and medicinal plants were collected, if available, through convenience sampling in Kaiti Division from July to August 2011. Five LGV, two wild fruits, and the leaves of six medicinal plants (Table 1) were harvested and identified by Patrick Maundu, a botanist at the National Museums of Kenya. Voucher specimens were deposited at the East African Herbarium in Nairobi and at McGill University.

Following collection all plant samples were washed with running water and air-dried and the fruits subsequently stored frozen (−6 to −18°C) and LGV and medicinal plants stored at 3 to 5°C until transfer to the University of Nairobi for freeze-drying within two weeks of collection. Freeze-dried plant samples were transferred to McGill University, Montreal, Canada, and stored in darkness at −20°C prior to extraction.

### 2.5. Plant Extractions

Freeze-dried LGV and medicinal plant leaves were ground using a Thomas Wiley Mini-Mill (Thomas Scientific) and a 20-mesh sieve. Flesh and peels of freeze-dried wild fruits were ground together using a mortar and pestle until the particles passed through the 20-mesh sieve. Ground plant samples were extracted using 80% ethanol according to the methodology described by Spoor et al. [24]. Nitrogen evaporation and freeze-drying were subsequently used to remove any remaining solvent with extracts stored in a desiccator at −20°C until experimental use.

### 2.6. Total Phenolics

To assess total phenolics, the Folin-Ciocalteu procedure was performed as described by Waterhouse [25]. Absorbance was read at 765 nm with a Synergy HT Multi-Mode Microplate Reader (Bio-Tek Instruments, Inc.). The mean blank value was subtracted from the standard and extract values. Total phenolic content was calculated based on a gallic acid standard curve and expressed as milligrams gallic acid equivalents (GAE) per gram of dry extract.

### 2.7. ORAC Assay

The ORAC assay was performed as previously described [26]. The change in fluorescence intensity over time was used to build the fluorescence decay curve compared to the decay curve of Trolox [27]. The final ORAC results were calculated as *μ*M Trolox equivalents (TE) per *μ*g of dry extract.

### 2.8. Cell Culture

All cell culture reagents were obtained from Wisent Inc. unless otherwise indicated. A human acute monocytic cell line (THP-1, ATCC) was cultured in Roswell Park Memorial Institute (RPMI) 1640 media supplemented with 10% fetal bovine serum, 0.05 mM beta-mercaptoethanol (Sigma-Aldrich Corp.), and 100 *μ*g/mL penicillin and 100 *μ*g/mL streptomycin in a 37°C humidified environment with 5% CO_2_. Cells were passed when they reached a cell density of approximately 8.0 × 10^5^ cells/mL.

### 2.9. Cell Viability and Cytotoxicity

To ensure that plant extract concentrations were not toxic towards the THP-1 monocyte cells used in the TNF-*α in vitro* assay, a Vi-CELL Cell Viability Analyzer (Beckman Coulter Inc.) was used to determine cell viability using a trypan blue exclusion assay. Toxicity was tested to determine the highest nontoxic concentrations up to 100 *μ*g/mL, the highest concentration tested in the TNF-*α in vitro* assay. If reduced cell viability was demonstrated (less than 80% viability), further tests were performed at 25, 50, and/or 75 *μ*g/mL. Plant extracts, appropriately diluted in 80% EtOH, were added to THP-1 monocyte cells (3.0 × 10^5^ cells/mL) in 12-well cell culture plates. A vehicle control, 80% EtOH, and a cell blank were also included in separate wells. The plate was then incubated at 37°C with 5% CO_2_ for 22 hours, which was equivalent to the total incubation period in the TNF-*α in vitro* assay.

Plant extracts that showed toxicity with the trypan blue assay were again solubilized in 80% EtOH and tested at 1, 10, 25, 30, 50, 75, and 100 *μ*g/mL using a CytoTox 96 Non-Radioactive Cytotoxicity Assay (Promega Corp.), which is a sensitive measure of cytotoxicity. Plant extracts and 50 M H_2_O_2_ (positive control for maximal LDH release) or EtOH (vehicle control) were added to THP-1 monocyte cells (2.0 × 10^5^ cells/mL) and incubated at 37°C with 5% CO_2_ for 22 hours. Following incubation, the plate was centrifuged at 2000 rpm for 10 minutes and then processed according to the manufacturer's protocol. Absorbance was read at 490 nm with a Synergy HT Multi-Mode Microplate Reader (Bio-Tek Instruments, Inc.). Absorbance values were blanked against the media control and percent viability was subsequently determined compared to the positive control (H_2_O_2_) and vehicle control (EtOH). The LDH values standardized to the EtOH control were used for calculating the median toxic dose (TC_50_), the test concentration calculated to reduce cell viability by 50%.

### 2.10. TNF-*α* Release Assay

An* in vitro* model of anti-inflammatory activity involving the inhibition of TNF-*α* release in THP-1 cells stimulated by LPS was performed according to Black et al. [28] with minor modifications. A water control, 80% EtOH vehicle control, and positive control (parthenolide, 10 *μ*g/mL and 1 *μ*g/mL, Sigma-Aldrich Corp.) were used. The plant extracts were tested at 10 *μ*g/mL and 50 *μ*g/mL or 100 *μ*g/mL (based on cytotoxicity data) for initial screening to identify active plants (THP-1 cell density of 1.01 × 10^5^ cells/mL). All controls and extracts were assayed in quadruplicate. Plant extracts that reduced TNF-*α* release by at least 20% (relative to LPS-stimulated cells treated with vehicle) were subsequently tested at 1, 10, 25, 50, 75, and/or 100 *μ*g/mL to characterize concentration-dependent effects and calculate IC_50_ values.

A Human TNF-*α* DuoSet ELISA kit (R&D Systems, Inc.) was used to quantify the amount of TNF-*α* released. The final TNF-*α* results were calculated as percent inhibition of TNF-*α* expression relative to the LPS-stimulated EtOH vehicle control and expressed as mean ± SEM (*n* = 11–32). To calculate the median inhibitory concentration (IC_50_) values, the means of the replicates for each concentration were plotted versus % inhibition (applying log transformations when warranted). The linear regression equation generated from the slope of the line was used to calculate the concentration at which inhibition was equal to 50% (i.e., IC_50_). The average of the three IC_50_ values was then taken to determine the overall IC_50_ value in *μ*g/mL and expressed as mean ± SEM (*n* = 3 proportional groups of data for each test concentration).

### 2.11. Statistics

Statistical differences between plant extract activities in the ORAC and TNF-*α* assays were identified using an ANOVA with Scheffé post hoc analysis. Significant differences in TNF-*α* inhibition by plant extracts compared to the vehicle or positive control were identified using an ANOVA with posthoc Dunnett's *t*-test (alpha = 0.05). Pearson correlation analysis was used to determine the relationship between antioxidant capacity, anti-inflammatory activity, and total phenolics content. Correlation between consensus scores and laboratory results was assessed using the Pearson correlation test. SAS version 9.2 was used for all statistical procedures.

## 3. Results

### 3.1. Dietary and Medicinal Plant Use

Traditional LGV and wild fruits comprised around half the vegetable and fruit consumption of the children in the study for the previous seven days' intake. The consumption of these LGV is likely higher during the wet seasons, as indicated by the percentage of children who consumed these plants (Table 1).* Amaranthus dubius*,* Vigna unguiculata*,* Cucurbita maxima*, and* Solanum scabrum* were among the most popular LGV for all participants (Table 1).* C. maxima* and* S. scabrum* were also cited in a medicinal context, such as treating malnutrition, stomach ache, or stomach ulcer. Wild fruit varieties were available in all seasons, which can be crucial during the dry season when fewer varieties of cultivated fruits are available.

Medicinal plants were used by 92% of the mothers to treat illnesses suffered by their children. Overall, participants reported using 97 medicinal plant preparations to treat 30 different symptoms; of these, 31 plants were used to treat the 8 specific symptoms targeted during interviews. Based on these results, 6 commonly used medicinal plants and one LGV used medicinally were collected. Consensus values were calculated for these seven plants, with* Z. chalybeum* and* O. americanum* showing the highest consensus (Table 1).

### 3.2. Total Phenolics and Antioxidant Activity

In general, medicinal plant extracts contained more phenolic metabolites and demonstrated stronger antioxidant activity than the LGV and wild fruits (Table 1). Three medicinal plants,* M. indica*,* P. guajava*, and* O. americanum*, had the highest phenolic content and greatest antioxidant activities, more than ten times the values obtained for wild fruit extracts. LGV extracts were richer in phenolic antioxidants than wild fruits but poorer than medicinal plants.The exception was* S. scabrum*, an LGV, which had the highest phenolic and ORAC scores among food plants (Table 1).

Total phenolic content and antioxidant activity among all plant extracts demonstrated a significant positive correlation (*P* < 0.0001, *r* ^2^ = 0.938, Pearson correlation analysis) but neither parameter correlated significantly with ethnobotanical consensus values.

### 3.3. Cell Viability

Six plants,* A. dubius*,* A. indica*,* C. maxima*,* O. americanum*,* S. scabrum*, and* V. unguiculata*, reduced cell viability by more than 20% at 100 *μ*g/mL as indicated by both cell viability and cytotoxicity assays. After establishing concentration-dependent toxicity, we calculated the TC_50_ for these extracts and determined their highest nontoxic concentrations for use in the TNF-*α* assay (Table 2). The remaining seven extracts appeared nontoxic at 100 *μ*g/mL, the maximum concentration subsequently administered to THP-1 cells.

### 3.4. TNF-*α* Inhibition

**Figure 1:**
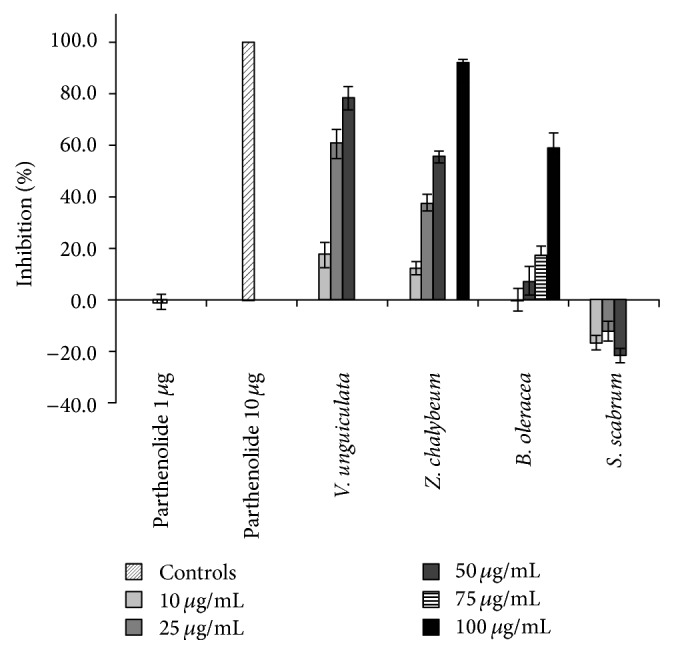
Inhibition of TNF-*α* expression in THP-1 monocyte cells by selected plant extracts. Parthenolide, a potent anti-inflammatory compound, was the positive control. Results shown as the mean and the error bars as the SEM (*n* = 11–32).

Both medicinal plants and LGV were among the most effective inhibitors of LPS-induced TNF-*α* release in THP-1 cell cultures.* A. dubius*, an LGV, was the most potent extract, with a IC_50_ of 9 *μ*g/mL, followed by* O. americanum*,* V. unguiculata*, and* Z. chalybeum* with IC_50_ values of 16, 27, and 47 *μ*g/mL, respectively (Table 2 and Figure 1).* B. oleracea*,* O. gratissimum*,* A. indica*, and* M. indica* showed a weaker dose response yet nonetheless produced significant inhibition at their highest test concentrations (relative to LPS-stimulated cells treated with vehicle) (data not shown).

Neither fruit extract significantly affected observed TNF-*α* levels following LPS stimulation. Demonstrating a potential proinflammatory effect,* S. scabrum*, an LGV, significantly increased TNF-*α* expression at all test concentrations (10, 25, and 50 *μ*g/mL; *P* < 0.05; Figure 1).

We observed no correlation between inhibition of TNF-*α* release and phenolic content or antioxidant activity. However, a significant negative correlation (*r* ^2^ = 0.7639, *P* = 0.0228) was found between consensus and IC_50_ results for anti-inflammatory activity. The negative correlation indicates that as IC_50_ values decrease (i.e., anti-inflammatory activity increases), the consensus over plant use increases. The correlation was based on complete data for six plants with both consensus and IC_50_ values (Tables 1 and 2).

## 4. Discussion

Young children in rural Kenya are at high risk of developing kwashiorkor, a developmentally debilitating malnutrition condition associated with edema, oxidative stress, chronic inflammation, and fatty liver [3, 4]. Wild plants, whether used as food or medicine, not only provide nutritional benefits but also often contain antioxidant and anti-inflammatory phytochemicals that when incorporated into diet may help prevent or manage kwashiorkor. After interviewing mothers with at least one child under the age of five years, we identified seven food plants and six medicinal plants commonly consumed by children, with* in vitro* analyses subsequently revealing that the majority of extracts exhibited significant antioxidant and anti-inflammatory potential.

The medicinal plants,* M. indica*,* P. guajava*, and* O. americanum*, had the greatest total phenolic content and antioxidant activity, corroborating previous reports of potent antioxidant activity in the leaves of these plants [29–31]. Several of the LGV demonstrated strong antioxidant and/or TNF-*α* inhibiting activity that was comparable to, or greater than, the activities of the medicinal plants.* S. scabrum*, one of the African nightshade species, was the LGV with the greatest total phenolic content and antioxidant activity. It is often used as an LGV and medicinal plant in Kenya and in other African countries [32]. Other studies corroborate the species' potent antioxidant activity [33, 34].

*A. dubius* and* V. unguiculata* leaves are particularly important as they were the most commonly eaten LGV among interviewed mothers and children (Table 1).* A. dubius*, the most potent inhibitor of TNF-*α* release among the tested plants, not only is nutritionally rich [35] but also has shown antioxidant and anti-inflammatory activities in previous studies [36]. While the seeds of* V. unguiculata* similarly display anti-inflammatory and antioxidant activities [17, 37], this is the first report of potent anti-inflammatory activity in the leaves. Since these two species of LGV are also widely eaten across Kenya [21], the pertinence of our results may extend beyond the region of this study.

The flesh and peels of the two wild fruits,* E. japonica* and* V. payos*, showed relatively low levels of phenolic metabolites, antioxidant activity, and anti-inflammatory potential. Our results do not support a previous study reporting significant antioxidant activity in both the flesh and peels of* V. payos* [38]. The discrepancy may be due to differences in the variety or maturation stage of* V. payos* samples or in plant extraction protocols and antioxidant assays. The seeds of* E. japonica* have been tested more widely and show greater antioxidant and phenolic content than the flesh and peels [39]. However, both fruits likely contribute to the overall health of children due to their vitamin content, specifically vitamins C and beta-carotene [19, 40].

Medicinal plants are an important part of the local culture in Makueni County and were used for treating illnesses in children by 92% of the interviewed mothers. Also several plants, including* O. americanum* and* P. guajava*, were not only used when the child was sick but also given as a general digestion aid [41]. Additionally,* Z. chalybeum* is added to tea for flavour and can be taken to improve appetite or provide energy [41]. The use of the medicinal plants to treat childhood illness and plants given to children for health in general, moreover, may be important in the context of kwashiorkor if inflammation is involved in the etiology.

High consensus can identify plants that are well known and commonly used, which may be based on efficacy for treating illness; thus it would be expected to correlate with biological efficacy [42]. Of the identified medicinal plants, informant consensus was strongest for* Z. chalybeum* and* O. americanum*, species that demonstrated significant antioxidant and anti-inflammatory activity. Given the focus of inflammation among the symptoms targeted during interviews, moreover, the significant positive correlation between consensus values and anti-inflammatory activity suggests that mothers are selecting not only active medicinal species but also the more potent ones. With these analyses limited by the targeted nature of the interview questions and the number of species collected in this study, a more comprehensive assessment of ethnobotanical knowledge among Kenyan mothers is warranted.

The provision of identified species by mothers to children, together with low toxicity of extracts observed in cell culture, suggests that the plants are well tolerated, at least at low concentrations. However, several of these species (or related taxa) are known to contain toxic metabolites of potential concern, particularly when dealing with young children.* S. scabrum*, for example, is a member of the* Solanum nigrum* complex (black nightshades), which contains solanine and related toxic glycoalkaloids [43]. Unlike poisonous and often indistinguishable European nightshades [44], edible African nightshades are widely consumed for their nutritional and medicinal value, collected from the wild, and sold in markets [19]. Since the phylogeny and phytochemistry of Africa's >30 wild* Solanum* species remain unclear, the leaves can be boiled or steamed prior to consumption, as this will remove the toxin [43].

Many* Zanthoxylum* species (Rutaceae) also contain toxic alkaloids [45–48] that warrant caution. For* Z. chalybeum*, both ethnobotanical and experimental data indicate that the species in this study is relatively nontoxic [49].* A. indica* was mildly cytotoxic in our study, as observed in brine shrimp [50]. Other identified species, such as* Amaranthus* spp. and* C. maxima*, have not demonstrated toxicity in previous studies [51]. Overall, while the studied medicinal plants, LGV, and fruits appear to pose limited risk to children, the potential for adverse effects persists, particularly with high doses or concomitant pharmacotherapies.

Additional epidemiological studies are needed to assess the potential for a diverse diet rich in vegetables and fruits with anti-inflammatory and antioxidant properties to improve health outcomes and reduce the risk of kwashiorkor development, with a consideration for potential toxicity. Local edible and medicinal plants, despite their accessibility and potential benefits, have yet to be studied in terms of either their specific impacts on kwashiorkor or their more broad contributions to child nutrition and health in rural Africa.

## 5. Conclusion

Beyond antioxidant activity, several of the studied medicinal plants and leafy green vegetables possess promising anti-inflammatory potential. Since mothers appear to select the more active plant species as food and medicine for their children, our results demonstrate both the cultural viability of these plants as potential foci for local food security and child health initiatives as well as the scientific and practical utility of linking biodiversity, ethnobotany, and pharmacology in search of viable and evidence-based interventions to address community health.

## Figures and Tables

**Table 1:** The species, consumption, antioxidant activity, and consensus values for the collected Kenyan plants.

Family name	Plant species^1^	Local name^2^	Type of plant	Consumption % during wet season^3^	Total phenolics^4^ Mg GAE/g ± SEM (*n* = 9–15)	ORAC activity^5^ *μ*M TE/*μ*g ± SEM (*n* = 6–10)	Consensus value^6^
Meliaceae	*Azadirachta indica *A. Juss.	Mairobaini	Medicinal^7^		78 ± 1^E^	1761 ± 243^CDEF^	0.0946
Amaranthaceae	*Amaranthus dubius* Mart. ex. Thell.	Muchicha	LGV	90%	39 ± 1^G^	928 ± 43^EF^	
Brassicaceae	*Brassica oleracea* L.	Sukuma	LGV	42%	48 ± 2^FG^	1184 ± 78^DEF^	
Cucurbitaceae	*Cucurbita maxima* Duchesne.	Matu ma malenge	LGV	65%	24 ± 1^H^	447 ± 71^F^	0.0170
Rosaceae	*Eriobotrya japonica* (Thunb.) Lindl.	Ndunda	Wild fruit	19%	14 ± 2^HI^	411 ± 15^F^	
Anacardiaceae	*Mangifera indica* L.	Maembe	Medicinal		337 ± 3^A^	5940 ± 632^A^	0.0351
Lamiaceae	*Ocimum americanum *L.	Mutaa	Medicinal		136 ± 3^C^	3190 ± 163^BC^	0.1264
Lamiaceae	*Ocimum gratissimum* L.	Mukandu	Medicinal		86 ± 2^DE^	1594 ± 168^CDEF^	0.0591
Myrtaceae	*Psidium guajava* L.	Mavela	Medicinal		258 ± 1^B^	3929 ± 411^B^	0.0757
Solanaceae	*Solanum scabrum* Mill.	Kitulu	LGV	55%	92 ± 3^D^	2675 ± 115^BCD^	
Fabaceae	*Vigna unguiculata* (L.) Walp.	Matu ma nthooko	LGV	73%	54 ± 3^F^	1233 ± 116^DEF^	
Verbenaceae	*Vitex payos* (Lour.) Merr.	Muu	Wild fruit	24%	7 ± 1^I^	179 ± 8^F^	
Rutaceae	*Zanthoxylum chalybeum* Engl.	Mukenea	Medicinal		92 ± 2^D^	2414 ± 117^CDE^	0.1598

^1^Species and author names from The Plant List database. ^2^Name of plants in Kikamba language. ^3^The percentage represents the average of the two wet seasons. A FFQ was used to obtain the dietary data. ^4^Values for total phenolics were calculated as milligrams gallic acid equivalents (GAE) per gram dry extract. ^5^Values for ORAC were calculated as *μ*M Trolox equivalents (TE) per microgram dry extract. ^6^Consensus values for medicinal plant use by the mothers. ^7^All medicinal plants were used, collected, and analyzed as leaves. ^A–I^Superscripts represent statistical differences between plant species at *P* < 0.05 using an ANOVA with Scheffé post hoc analysis.

**Table 2:** Cytotoxicity and inhibition of tumour necrosis factor-alpha production by plant extracts in THP-1 monocytes.

Plant species	TC_50_^1^ (*μ*g/mL ± SEM)	Highest test concentration (*μ*g/mL)^2^	IC_50_ (*μ*g/mL ± SEM)^3^
Medicinal plants			
* A. indica *	204 ± 75	50	58 ± 2^C^
* M. indica *		100	169 ± 6^E^
* O. americanum *	80 ± 16	25	16 ± 1^AB^
* O. gratissimum *		100	112 ± 6^D^
* P. guajava *		100	
* Z. chalybeum *		100	47 ± 1^BC^
Leafy green vegetables			
* A. dubius *	84 ± 3	25	9 ± 1^A^
* B. oleracea *		100	111 ± 11^D^
* C. maxima *	58 ± 30	50	131 ± 4^D^
* S. scabrum *	114 ± 16	50	
* V. unguiculata *	102 ± 9	50	27 ± 5^ABC^
Wild fruits			
* E. japonica *		100	
* V. payos *		100	

^1^TC_50_ is the toxic dose, or the concentration calculated to reduce cell viability by 50%. *n* = 4 replicates for each test concentration. ^2^Highest concentration tested that reduced cell viability by less than 20%. ^3^ *n* = 3 proportional groups comprised of the results for each test concentration. ^A–I^Superscripts represent statistical differences between plant species at *P* < 0.05 using an ANOVA with Scheffé post hoc analysis.
